# An open deep learning-based framework and model for tooth instance segmentation in dental CBCT

**DOI:** 10.1007/s00784-025-06578-w

**Published:** 2025-09-25

**Authors:** You Zhou, Yan Xu, Basel Khalil, Andrew Nalley, Mihai Tarce

**Affiliations:** 1 Periodontology & Implant Dentistry, Faculty of Dentistry, The Prince Philip Dental Hospital, The University of Hong Kong, 34 Hospital Road, Sai Ying Pun, Hong Kong; 2 Applied Oral Sciences & Community Dental Care, Faculty of Dentistry, The Prince Philip Dental Hospital, The University of Hong Kong, 34 Hospital Road, Sai Ying Pun, Hong Kong; 3 Department of Stomatology, Nanjing Drum Tower Hospital, The Affiliated Hospital of Nanjing University Medical School, No. 321 Zhongshan Road, Nanjing, Jiangsu China

**Keywords:** Artificial intelligence, Cone-beam computed tomography, Instance labelling, Maxillofacial imaging, 3D slicer

## Abstract

**Objectives:**

Current dental CBCT segmentation tools often lack accuracy, accessibility, or comprehensive anatomical coverage. To address this, we constructed a densely annotated dental CBCT dataset and developed a deep learning model, OraSeg, for tooth-level instance segmentation, which is then deployed as a one-click tool and made freely accessible for non-commercial use.

**Materials and methods:**

We established a standardized annotated dataset covering 35 key oral anatomical structures and employed UNetR as the backbone network, combining Swin Transformer and the spatial Mamba module for multi-scale residual feature fusion. The OralSeg model was designed and optimized for precise instance segmentation of dental CBCT images, and integrated into the 3D Slicer platform, providing a graphical user interface for one-click segmentation.

**Results:**

OralSeg had a Dice similarity coefficient of 0.8316 ± 0.0305 on CBCT instance segmentation compared to SwinUNETR and 3D U-Net. The model significantly improves segmentation performance, especially in complex oral anatomical structures, such as apical areas, alveolar bone margins, and mandibular nerve canals.

**Conclusion:**

The OralSeg model presented in this study provides an effective solution for instance segmentation of dental CBCT images. The tool allows clinical dentists and researchers with no AI background to perform one-click segmentation, and may be applicable in various clinical and research contexts.

**Clinical relevance:**

OralSeg can offer researchers and clinicians a user-friendly tool for tooth-level instance segmentation, which may assist in clinical diagnosis, educational training, and research, and contribute to the broader adoption of digital dentistry in precision medicine.

**Supplementary Information:**

The online version contains supplementary material available at 10.1007/s00784-025-06578-w.

## Introduction

With the continuous development and widespread application of digital technology in modern medicine, digital dentistry has become mainstream. The key lies in the efficient acquisition and accurate identification of three-dimensional anatomical structures and detailed information in the oral and maxillofacial region [1, 2]. Cone-Beam Computed Tomography (CBCT), as an essential imaging tool in dentistry, offers significant advantages such as low radiation dose, high spatial resolution, and an optimized signal-to-noise ratio, enabling clear visualization of the complex anatomical relationships between teeth and jawbones through 3D reconstruction [3, 4]. The high-quality hard tissue images generated by CBCT significantly enhance the accuracy of clinical diagnosis, assist dentists in precisely locating pathological areas, formulating personalized treatment plans, and evaluating therapeutic outcomes [5, 6]. CBCT imaging has been widely applied in orthodontic treatment planning, orthognathic surgery assessment, preoperative placing of dental implants, and comprehensive diagnosis of various maxillofacial diseases, providing a solid technical foundation for precision medicine and individualized therapy [7–10].

While accurate tooth identification and segmentation from CBCT images—particularly at the instance level for individual teeth—remains technically complex, recent advances in artificial intelligence have substantially improved the feasibility of this task. Compared to the entire maxillofacial image in large FOV, individual teeth only occupy a very small proportion of spatial voxels, especially in the root apical regions, where anatomical structures are complex and variable, and grayscale values are similar to surrounding alveolar bone, making segmentation susceptible to imaging noise, uneven contrast, and underexposure [11]. A further challenge associated with segmentation is the complexity of anatomical structures, such as teeth occlusion, crowding, variation in number of teeth, and arch length [12–14]. Although teeth generally share similar morphology, their occlusal surfaces are complex, contours exhibit significant topological variation, and there are often no clear boundaries between adjacent teeth. Furthermore, restorative materials such as crowns, metal posts, and implants often generate artifacts in CBCT images, resulting in image detail loss and boundary blurring [15]. In addition, even slight movements of the patients during image acquisition may lead to image blurring or distortion, which affects segmentation accuracy [16]. Although these factors pose challenges, the growing application of AI-driven methods has shown promising capabilities in addressing these limitations.

Currently, tooth segmentation methods are mainly classified into manual segmentation and semi-automatic (SA) segmentation. Manual segmentation typically relies on experts annotating CBCT images slice by slice, which is not only experience-dependent but also time-consuming. Even for experienced dental specialists, completing a full tooth segmentation case may take 2 to 5 h [17]. SA segmentation methods rely on image processing techniques such as thresholding, region growing, and edge detection, using grayscale differences, pixel consistency, and structural boundaries to assist in segmentation [18–20]. These methods can produce relatively good results when applied to high-quality, well-structured images such as bone areas [21, 22]. However, they are highly susceptible to image noise and grayscale differences, making them less suitable for complex oral structures or significant anatomical variation [23, 24]. These limitations particularly occur in instance segmentation with multiple labels, highlighting the need for more automated solutions.

Artificial intelligence, particularly deep learning techniques, has advanced rapidly in recent years, with a growing number of studies applying it to automatic segmentation tasks in 3D medical imaging. Cui et al. [25] proposed a two-stage segmentation method called ToothNet, marking the first application of deep learning for tooth segmentation in CBCT images. This method utilized Mask R-CNN to extract tooth edge maps from the input images and fused them with the spatial features of the original images to enhance tooth region recognition [26]. Although this method made some progress, it still exhibited notable limitations, including misrecognition or misplacement of teeth in instance detection. Ezhov et al. [27] built upon the V-Net architecture to propose a coarse-to-fine segmentation framework for precise localization and segmentation of individual teeth [28]. The method processed CBCT scans at full resolution by first using a coarse segmentation model to predict tooth type and location, followed by a fine model that generated a probability map for the target tooth, thereby enabling instance-level detection and segmentation. This framework effectively improved spatial consistency and accuracy of segmentation. However, due to the large variety of tooth types and significant individual differences, the model suffered from classification inaccuracies on small-sample datasets, which limited its further improvements in generalization performance.

A recent systematic review indicates that current deep learning approaches for tooth segmentation in CBCT generally face the following major challenges [29]:

1. Due to the typical small target characteristic of teeth in CBCT images, it is difficult to process the entire image directly, so most methods adopt multi-stage segmentation approaches, starting from coarse to fine or large to small. However, these approaches accumulate errors at each stage, and the cropped images often lose the spatial relationships of anatomical features.
2. Most existing methods remain focused on semantic segmentation, distinguishing teeth from bones, while fewer studies target instance segmentation of individual teeth. However, the prediction results are generally poor, with high misidentification rates.
3. Although tooth segmentation has become a popular research topic, most research only reports results without releasing source code or pre-trained models, making study replication and objective evaluation impossible.
4. Currently, large-scale, high-precision public datasets with standardized annotations are scarce, making it difficult to achieve consensus on segmentation accuracy, consistency, and comparability.

This study aims to establish and implement a standardized annotation process, labeling key anatomical structures in CBCT images precisely to build a densely annotated instance segmentation dataset. Subsequently, using UNetR as the backbone network and incorporating the Swin Transformer and spatial Mamba modules for multi-scale residual feature fusion, the deep learning segmentation model OralSeg is designed and optimized to meet the requirements of tooth instance segmentation [30–32]. Finally, OralSeg is released publicly with a non-commercial license and integrates into the 3D Slicer imaging platform, offering an accessible solution for clinical dental education and related research, especially for dentists without previous experience in AI model training.

## Methods

### Study design

This study is divided into three main stages: data acquisition and image annotation, segmentation model training and performance evaluation, and finally the design and release of the 3D Slicer extension.

### Data acquisition

This study was conducted as a retrospective study. All data acquisition and research protocols were approved by the Institutional Review Board of The University of Hong Kong/Hospital Authority Hong Kong West Cluster (Approval No.: HKWC-2025-103) and the Ethics Committee of Nanjing Drum Tower Hospital, affiliated with Nanjing University Medical School (Approval No.: 2024-302-01). The study strictly followed the Declaration of Helsinki and relevant ethical guidelines to ensure the privacy and data security of participants.

The CBCT scans used in this study were collected from 100 patients who underwent scans for diagnostic or therapeutic purposes at the Department of Stomatology, Nanjing Drum Tower Hospital, between June 2019 and May 2024.

Participants were required to meet the following criteria:

1. Patients must be 18 years old or above.
2. Patients had a complete dentition or were partially edentulous.
3. Patients with restorations, crowns, and implants were included.
4. All CBCT scans were obtained using the same equipment (NewTom Giano, Verona, Italy) and a standardized scanning protocol with a field of view (FOV) of 13 cm × 10 cm.
5. The CBCT images provided anatomical details, including all teeth, jawbone, and craniofacial structures.

Exclusion criteria included:

1. Patients who were completely edentulous.
2. Patients with a history of maxillofacial tumors, fractures, trauma, large bone defects, or inflammatory lesions to avoid interference from pathology.
3. Images with motion blur and artifacts caused by patient movement during scanning.

Two trained and calibrated dentists independently screened the collected imaging data. In case of disagreements, a third dentist who received the same training made the final decision. Researchers removed all directly identifiable personal information (e.g., names, ages, genders, addresses, phone numbers) from the collected data, replacing these identifiers with unique codes to ensure participant anonymity and confidentiality. The acquired CBCT dataset comprises a volumetric matrix of 440 × 440 × 344 voxels with an isotropic in-plane resolution and an inter-slice spacing of 0.3 mm.

### Annotation and consistency

Two dentists manually annotated CBCT images using 3D Slicer, a free and open-source software for medical image processing and analysis. A total of 36 key anatomical structures were annotated, including 32 individual teeth (labeled according to the FDI tooth numbering system), the maxilla, the mandible, and the bilateral mandibular canals. All annotations were named using standardized terminology and a unified color-coding system. Initial coarse segmentation was performed using the DentalSegmentator extension [33] in 3D Slicer, followed by slice-by-slice verification and refinement using manual tools such as draw, erase, and scissors to obtain fine-grained segmentation results. These finely annotated images served as the ground truth for training the deep learning model.

To ensure annotation quality, 5 cases were randomly selected from the full dataset as a calibration set, and inter-expert consistency was evaluated using Dice similarity coefficient (DSC) and Intraclass correlation coefficient (ICC) for each label.

### Model architecture

**Fig. 1:**
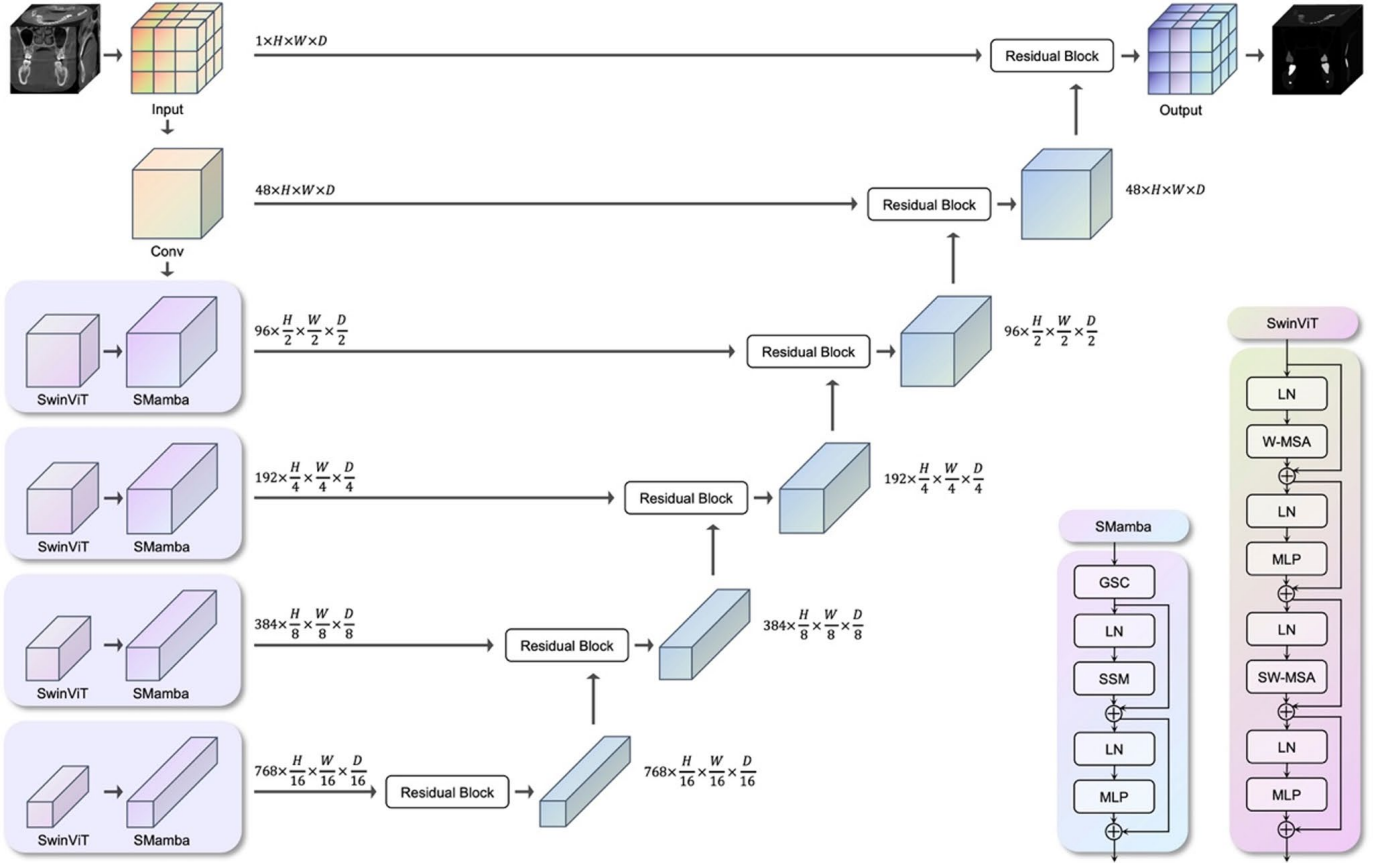
Overview of the OralSeg architecture. Each encoder scale integrates a Swin Transformer (SwinViT) and a Spatial Mamba (SMamba) module, while residual skip connections fuse multi-scale features in the decoding phase

The OralSeg network shown in Fig. 1 consists of three key modules: the Swin Transformer (SwinViT) module for extracting global image features, the spatial Mamba (SMamba) block for capturing spatial relationships and local details, and multi-scale residual skip connections for feature enhancement and information transfer during the decoding process. Specifically, at each feature extraction scale, OralSeg first applies the SwinViT encoder to perform global modeling of the CBCT image, then combines its output with the spatially oriented Mamba block to refine boundaries, details, and local structures.

The SwinViT partitions the input dental CBCT images into 3D patches of varying sizes and calculates self-attention within each patch using a volumetric shifting window mechanism, enabling context modeling across patches and 3D feature interactions within and between layers. The entire encoder comprises four stages, each using patches of size 2 × 2 × 2, with the feature dimension set to 8 and an embedding spatial size of 48, tailored to the characteristics of the single-modality CBCT images.

The Mamba module specializes in enhancing spatial structure modeling capabilities. This module employs two scales of 3D convolution operations (with kernel sizes of 1 × 1 × 1 and 3 × 3 × 3), where the smaller kernel emphasizes image details and the larger kernel captures broader spatial context. The outputs from the two convolution blocks are fused to enable joint modeling of local details and broader context. This process occurs concurrently in all three spatial dimensions, thereby enhancing the spatial integrity and coherence of the feature representation.

At each encoding stage, the features extracted by SwinViT and the spatial Mamba module are stacked, and spatial downsampling (decreasing the resolution by a factor of 2) is applied to maintain the encoder’s hierarchical structure. In the decoding phase, the network employs a multi-scale skip-connection mechanism to incorporate fused features from different encoding layers, which are then enhanced through modules containing residual blocks. After the decoding features from the previous stage are upsampled (increasing the resolution by a factor of 2), they are concatenated with the current stage’s skip-connected features, progressively restoring the image’s spatial resolution. The entire network adopts a typical U-shaped structure, ultimately generating a 3D segmentation probability map through a 1 × 1 × 1 convolution layer and a sigmoid activation function.

### Training

The training process in this research was conducted on a high-performance workstation with an NVIDIA A100 GPU, utilizing deep learning frameworks including PyTorch (2.6.0), Medical Open Network for AI (MONAI, 1.4.0), and Linear-Time Sequence Modeling with Selective State Spaces (Mamba-SSM, 1.2.0). All anonymized CBCT data were randomly split into 70% for training, 20% for validation, and 10% for testing, and each dataset was paired with its respective annotation file (Figure S1). To enhance the robustness and generalizability of the model, multiple data augmentation techniques were applied before training, including random flips, rotations, intensity scaling and shift, simulating morphological variations in clinical images. For image preprocessing, following standard deep learning image procedures, the voxel intensities of CBCT images were normalized to a range of 0 to 1, unifying the image’s dynamic range. All experiments followed a same training protocol. We sampled patches through random 3D cropping with a patch size of 64 × 64 × 64 and a batch size of 1, optimized the networks using DiceCELoss (Dice + cross-entropy composite), initialized the learning rate at 1 × 10⁻⁵, and trained for 2,500 epochs for each dataset. Regarding the optimizer, AdamW was used in conjunction with a cosine annealing learning rate scheduler (warmup_cosine), achieving smoother gradient updates during the initial training phase and enhancing later-stage model generalization to reduce overfitting. This strategy stabilized model training by initially introducing a warm-up phase and then gradually lowering the learning rate.

In addition, to further validate the effectiveness of each component in the proposed structure (OralSeg), this study designed and conducted systematic ablation experiments. By removing or replacing key components in the network architecture, models with an independent Swin Transformer module and with spatial Mamba were created. The impact of these modifications on performance was studied to clarify each module’s specific contribution to improving tooth segmentation accuracy. Meanwhile, comparisons were made between the fine-tuned model OralSeg and both the backbone SwinUNETR [34, 35] as well as the classic 3D U-Net [36] to verify its performance in tooth instance segmentation tasks. All models were evaluated on an independent test CBCT dataset that was excluded from both the training and validation process, ensuring the objectivity of results and effectively reflecting the model’s generalizability.

### Evaluation metrics

In this study, evaluation metrics (EM) for the model included the Dice similarity coefficient, Intersection over Union (IoU), accuracy, precision, sensitivity, and F1-score. Each class is individually calculated first and then the mean of evaluation metrics is obtained by averaging across all classes:

$$\:mEM=\frac{1}{N}\sum\:_{i=1}^{N}{EM}_{i}$$

where 𝑁 is the total number of classes.

### Extension

The Oral Implant Tools extension for 3D Slicer (version 5.8.1) uses the model to automatically segment CBCT scans [37]. Since our model performs best on CBCT scans having similar parameters to our homogenous training dataset, the extension ensures the spacing of the input volume matches our dataset, resampling it if necessary. It can perform both instance and semantic segmentation (by merging the tooth and bone instances). The source code for the extension will be made available at https://github.com/mihaitarce/SlicerOralImplantTools.

### Statistical analysis

Comparisons of Dice across models were conducted using linear mixed-effects models at the case × label level, and results are reported as estimated marginal means on the original scale with 95% CIs. In addition, we retained macro-averaged Dice as descriptive statistics only (mean ± SD). A repeated measurement one-way ANOVA with the Geisser-Greenhouse correction was chosen to compare segmentation results. For comparisons across anatomical categories within the same model, we used ordinary one-way ANOVA followed by Tukey’s multiple comparisons. Two-sided *p* < 0.05 was considered statistically significant.

## Results

A total of 100 CBCT images were densely annotated. Dataset characteristics including dentition status, third-molar prevalence, and dental restorations are summarized in Table S2. The DSC and ICC were used to assess inter-annotator consistency between two dentists for each label. The mean ICC was 0.96, indicating excellent agreement and supporting the reliability of the dataset. Peak GPU memory consumption was 21.85 GB during training and 7.58 GB during inference, measured under our experimental setup.

**Fig. 2:**
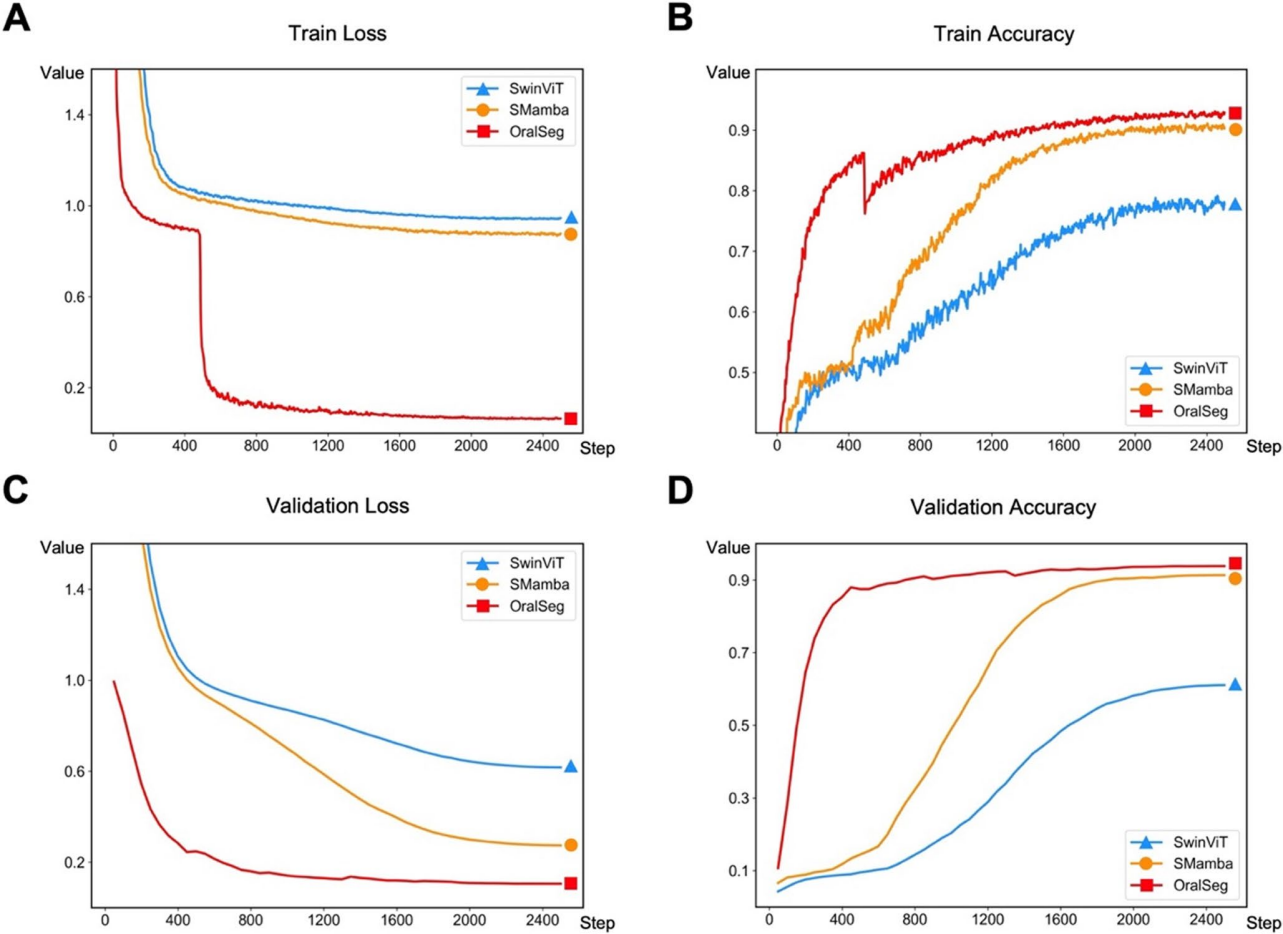
Training and validation performance of model and ablation models. (**A**) Training loss curves. (**B**) Training accuracy curves. (**C**) Validation loss curves. (**D**) Validation accuracy curves for SwinViT (blue), SMamba (orange), and OralSeg (red)

By analyzing training loss, training accuracy, validation loss, and validation accuracy, Fig. 2 presents the performance comparison of three deep learning structures (SwinViT, SMamba, and OralSeg) under different ablation configurations. All three models exhibited a rapid decrease in training loss with the progression of training steps. OralSeg quickly reached a low loss after around 800 training steps and remained below 0.1. SwinViT and SMamba exhibited relatively high training loss in the early stages with a slower decline later, indicating that the model had converged but faced higher optimization difficulty (Fig. 2A). Comparing model training accuracy, SwinViT showed continuous improvement during training but lagged behind the other two models. SMamba showed a more stable growth trend and eventually reached results close to OralSeg which showed rapid improvement in training accuracy during the early stages and ultimately achieved higher levels. It converged faster and demonstrated greater efficiency in feature extraction and gradient optimization (Fig. 2B). Figure 2C and D showed the comparative performance of the models on the validation dataset. The trend of validation loss changes was similar to training loss, but the performance differences on the validation set were more significant. The validation loss for all three models decreased gradually and eventually converged, with OralSeg showing a smoother decrease in loss. Furthermore, OralSeg’s validation accuracy increased rapidly and stabilized, demonstrating better generalization ability. SMamba’s accuracy on the validation set increased steadily and eventually reached a high level. SwinViT showed slower accuracy improvement, particularly in the early training stages, with validation accuracy rising more slowly compared to SMamba and OralSeg.

**Fig. 3:**
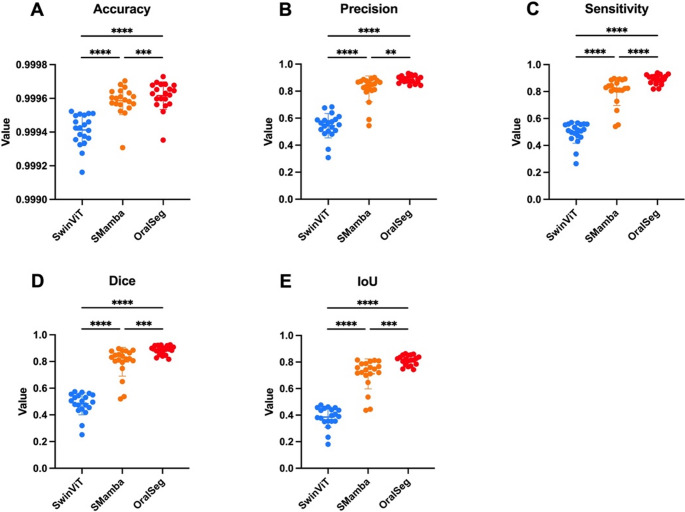
Segmentation performance comparison of model and ablation models using different metrics. (**A**) Accuracy of SwinViT, SMamba and OralSeg. (**B**) Precision of SwinViT, SMamba and OralSeg. (**C**) Sensitivity of SwinViT, SMamba and OralSeg. (**D**) Macro-average Dice of SwinViT, SMamba and OralSeg. (E) IoU of SwinViT, SMamba and OralSeg. Data are presented as mean ± SD. *p < 0.05, **p < 0.01, ***p < 0.001, and ****p < 0.0001 indicate significant differences versus the two groups

OralSeg achieved the highest overall Dice (0.8972, 95% CI 0.8707–0.9188), followed by SMamba, while SwinViT was substantially lower (Table 1). Pairwise contrasts on the original scale showed the same trends (Figure S2). Figure 3 and Table S1 show the mean, standard deviation, and statistical differences of the three models after the ablation experiments across five evaluation metrics: accuracy, precision, sensitivity, macro-average Dice, and IoU. All models exhibited high accuracy (Fig. 3A). In terms of precision, OralSeg and SMamba performed better, achieving 0.8876 ± 0.0265 and 0.8174 ± 0.0934 respectively, indicating that the introduction of the spatial Mamba module effectively improved the accurate recognition of tooth and bone structures, reducing false positive predictions (Fig. 3B). In terms of sensitivity, compared to the other two structures, OralSeg exhibited higher sensitivity at 0.8910 ± 0.0338, showing that the fusion of SwinTransformer and Mamba significantly enhanced the model’s ability to segment fine structures (Fig. 3C). OralSeg performed better than SwinViT and SMamba on both the macro-average Dice and IoU, achieving 0.8848 ± 0.0322 and 0.8132 ± 0.0363. Moreover, compared to SwinViT, the segmentation results with the spatial Mamba module were more stable and focused (Figs. 3D and E). The results indicated that the integration of the spatial Mamba module significantly enhanced the model’s understanding of spatial structures and strengthened its ability to extract foreground regions.

**Table 1:** Estimated marginal means (EMM) of dice with 95% confidence intervals by ablation models

	EMM_Dice	CI_low	CI_high
SwinViT	0.1389	0.1107	0.1729
SMamba	0.8392	0.8011	0.8712
OralSeg	0.8972	0.8707	0.9188

Table 2 further demonstrates the specific performance of the three models on the validation set. The presence of crowns and restorations may affect segmentation outcomes. In the validation images, a total of 33 specific categories were marked as empty due to tooth loss, restorations, or implants, meaning that the label existed but did not contain any voxels. The distribution is summarized in Table 2S to provide additional context on dental status. SwinViT correctly identified only 4 of these categories, while SMamba identified 15 categories, demonstrating the significant advantage of the spatial Mamba module in capturing long-range spatial information. In contrast, OralSeg performed the best, successfully identifying 30 categories. Notably, in the 3 false identified categories, two errors were related to fixed bridge restorations from tooth 14 to 17 in one patient, and there were also high-intensity artifacts present. OralSeg segmented the bridge of 15 and 16 as separate teeth. Moreover, during segmentation, the model may fail to predict certain labels, leading to missing segmentation. The results showed that SMamba and OralSeg significantly reduced the occurrence of prediction loss and only 4 labels failed to predict. Compared to SwinViT which missed 82 labels, the performance of these two models was more stable and they handled complex cases better, reducing false negatives and omissions.

**Table 2:** Performance comparison of model and ablation models in correct identify, false identify, and missing segment

	Identify	False Identify	Missing Segment
SwinViT	4	29	82
SMamba	15	18	4
OralSeg	30	3	4

**Fig. 4:**
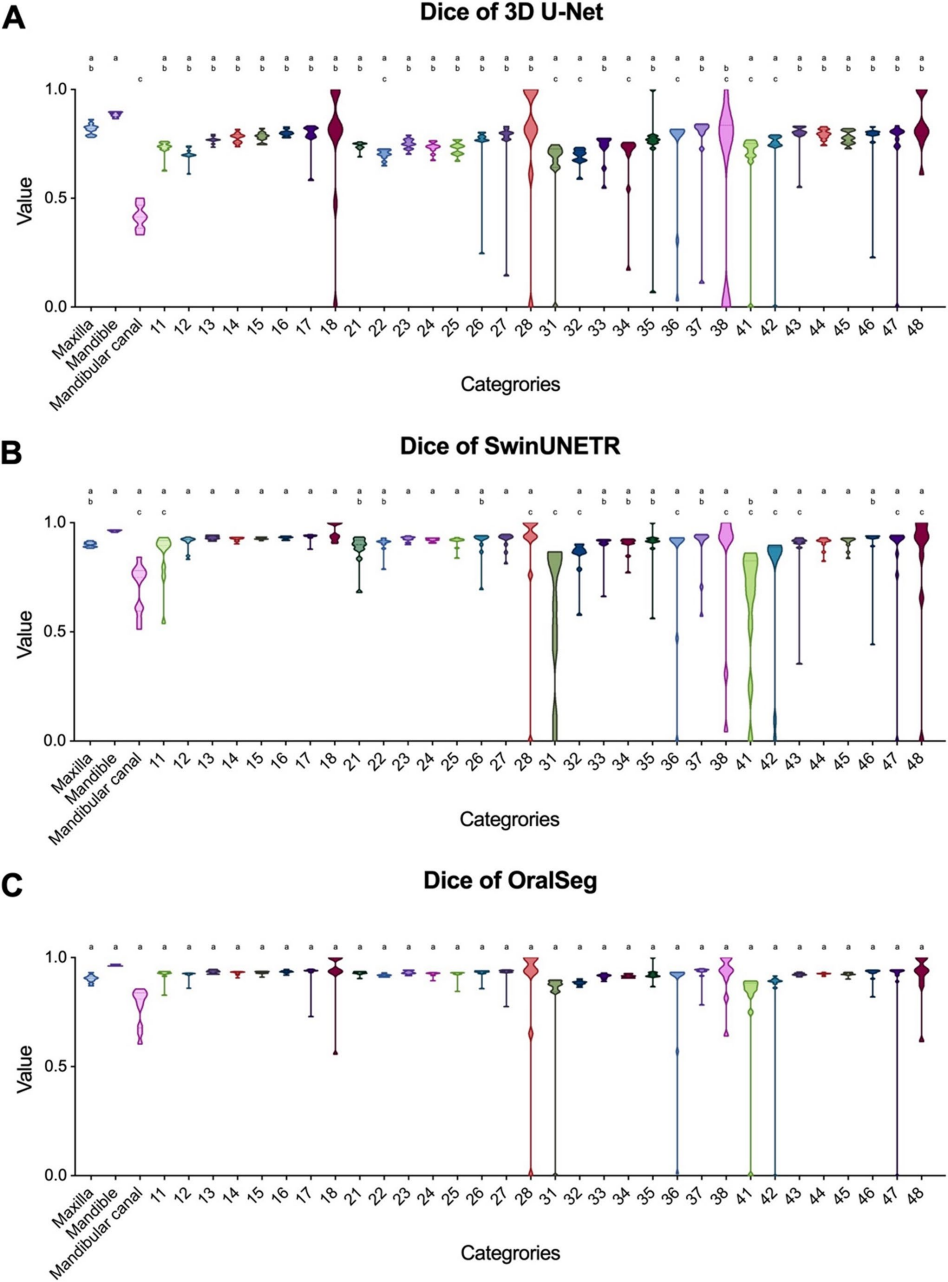
Dice for each label based on the segmentation results of 3D U-Net, SwinUNETR, and OralSeg models on the test dataset. (**A**) Dice of 3D U-Net (**B**) Dice of SwinUNETR. (**C**) Dice of OralSeg. Data are presented as mean ± SD. p-values less than 0.05 were considered significant. Groups sharing the same letter indicate no statistically significant difference between them

The Dice distribution for instance segmentation performed by SwinUNETR, OralSeg, and 3D U-Net on test images was shown in Fig. 4, with statistical differences between categories marked by letters. 3D U-Net performed relatively poorly, with lower Dice across all categories, particularly when processing fine structures such as the mandibular nerve canal and lower anterior teeth (Fig. 4C). However, for structures with higher voxel proportions, such as the maxilla and mandible, 3D U-Net showed good segmentation performance. The results indicated that 3D U-Net faced bottlenecks when segmenting small structures and needed further optimization to improve its segmentation performance on fine details. SwinUNETR demonstrated stable performance across most categories and can effectively extract image features. However, segmentation of lower anterior teeth showed clear shortcomings, with lower Dice coefficients and larger variability in the segmentation results (Fig. 4A), indicating the model’s limitations in handling small targets or complex anatomical areas. OralSeg showed the best stability and accuracy in segmentation tasks across most categories, especially in segmenting complex and small structures (Fig. 4B). The Dice of OralSeg was shown in Table 3 (0.9121, 95% CI 0.8707–0.9188) and the distribution was more uniform, with no significant statistical differences in segmentation performance across categories. Table 4 provides a further comparison of other evaluation metrics on the test set. Similar to Dice, OralSeg obtained the best results, with a precision of 0.8943 ± 0.9288, a sensitivity of 0.9005 ± 0.0327, an IoU of 0.8316 ± 0.0305 and a F1-score of 0.9003, achieving a good balance across all evaluation metrics and showing greater adaptability and accuracy.

**Table 3:** EMM of dice with 95% CI of 3D U-Net, SwinUNETR, and OralSeg

	EMM_Dice	CI_low	CI_high
3D-UNet	0.7359	0.6919	0.7757
SwinUNETR	0.8834	0.8592	0.9038
OralSeg	0.9121	0.8943	0.9288

**Table 4:** Segmentation performance comparison of 3D U-Net, SwinUNETR, and OralSeg using different metrics

	3D U-Net	SwinUNETR	OralSeg
Accuracy	0.9991 ± 0.0001	0.9996 ± 0.0001	0.9996 ± 0.0001
Precision	0.7367 ± 0.0399	0.8755 ± 0.0477	0.9000 ± 0.0198
Sensitivity	0.7284 ± 0.0425	0.8620 ± 0.0554	0.9005 ± 0.0327
MacroDice	0.7296 ± 0.0423	0.8623 ± 0.0608	0.8983 ± 0.0264
IoU	0.5974 ± 0.0405	0.7874 ± 0.0721	0.8316 ± 0.0305
F1-score	0.7325	0.8687	0.9003

**Fig. 5:**
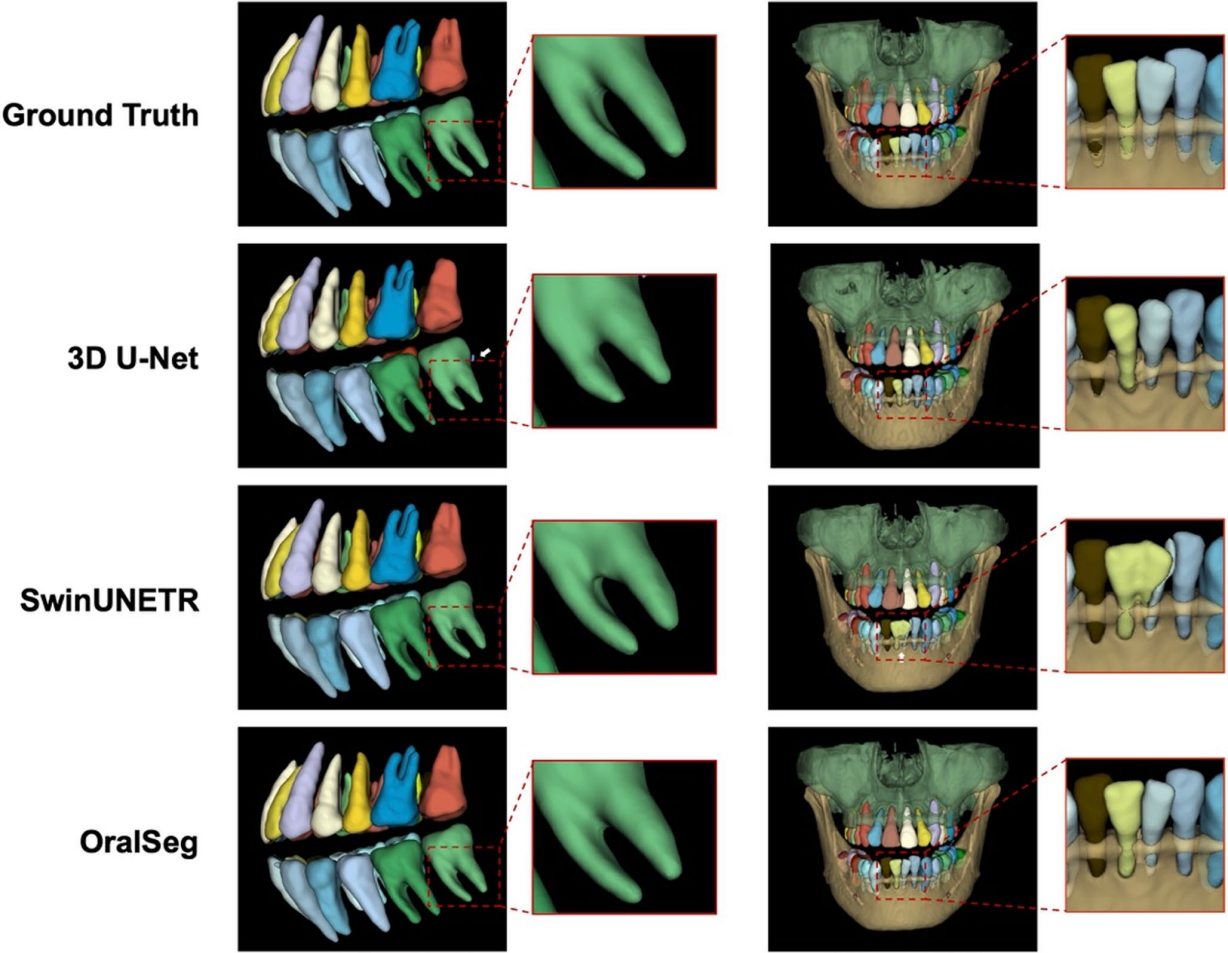
Visual comparison of instance segmentation results from 3D U-Net, SwinUNETR, and OralSeg. The first column demonstrated the segmentation of the upper and lower dental arches. The second column illustrated overall segmentation performance. Different color codes indicated different instance labels. White arrows highlighted typical missegmentation regions produced by the models

The 3D instance segmentation results are compared in detail with the Ground Truth in Fig. 5, including dentition and full-range CBCT scans. Color coding was used to distinguish different target structures, facilitating visualization and comparison. The manual annotations represented the ideal segmentation, where different structures such as individual teeth, maxilla, mandible, and mandibular canals were spatially independent and delineated. All teeth exhibited accurate morphological detail, including detailed occlusal surfaces, roots, interproximal contacts, and clearly defined boundaries between teeth and alveolar bone. Compared to Ground Truth, SwinUNETR captured the overall tooth morphology with smooth edges and performed well in extracting global structures. However, there was significant deformation and inaccurate segmentation in details such as the roots of the lower anterior teeth and interproximal contact point. For the mandibular canal, SwinUNETR can approximately locate its position but showed evident discontinuities along the canal wall. OralSeg’s segmentation aligned most closely with the Ground Truth, maintaining key anatomical details including tooth shape, neck, root furcation, apices, and mandibular canal integrity. Compared to SwinUNETR and 3D U-Net, OralSeg showed clearer contours in small and complex regions. In 3D U-Net, notable structural omissions or false segmentations were observed. Some teeth exhibit apical or marginal defects. Fusion or discontinuity was also observed at the boundary between the mandible and teeth. False segmentations occurred in some edentulous areas, particularly the third molars, often resulting in misidentification near the distal second molars or root zones. Furthermore, 3D U-Net often produced discontinuous or shifted segmentation, reflecting its limitation in identifying long and complex structures like the mandibular canal.

**Fig. 6:**
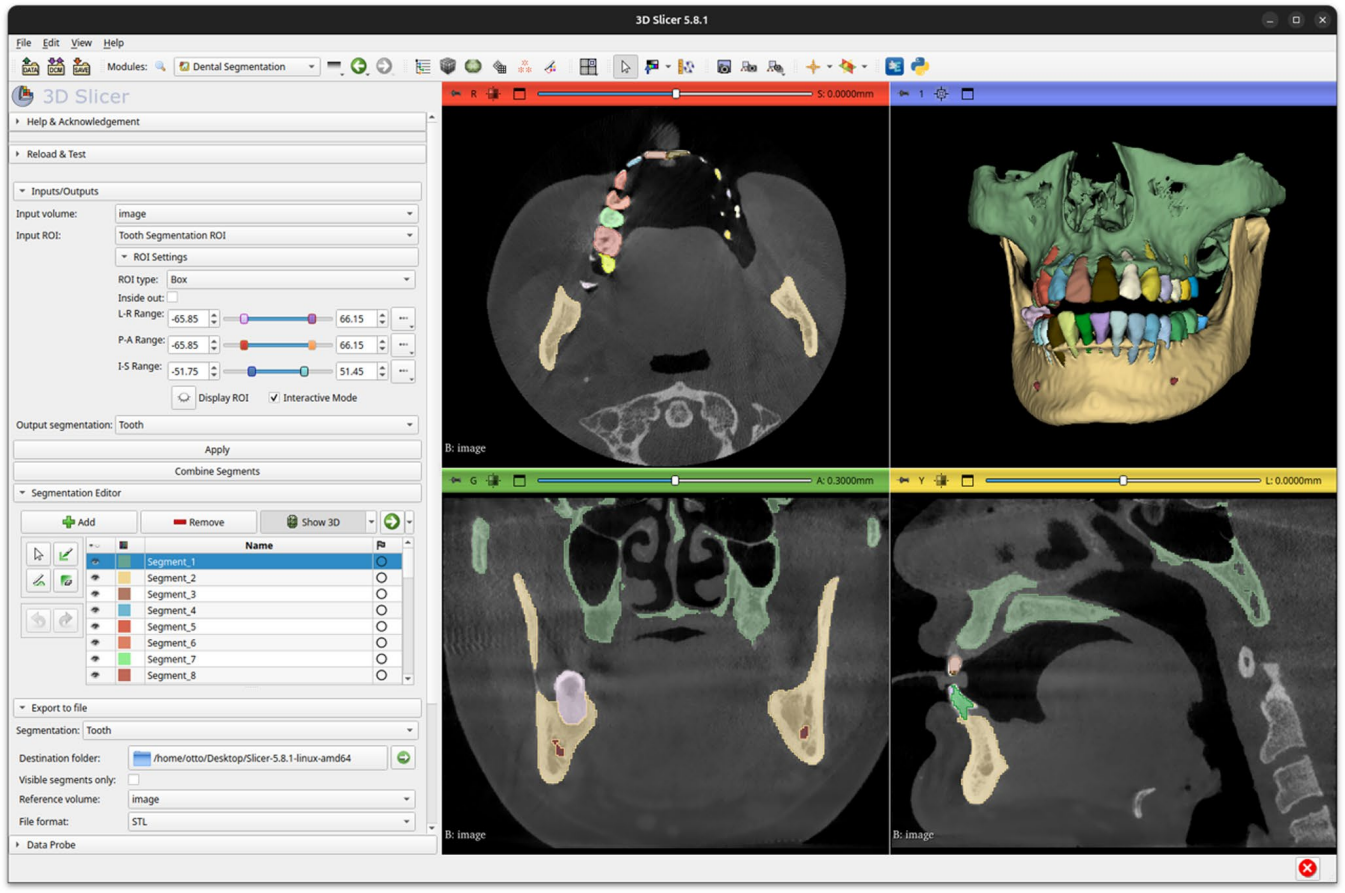
The user interface and segmentation results of the OralSeg extension in 3D Slicer

Figure 6 displays the user interface of the OralSeg extension in 3D Slicer. Users can adjust the ROI to adapt to varying computational capabilities, which enables flexible deployment and execution of segmentation tasks. Segmentation results can be exported in various medical image formats, facilitating clinical diagnosis or further analysis by dentists and enhancing its practicality and value in clinical and research settings. Development and testing were performed on Linux (Ubuntu 22.04) using 3D Slicer (version 5.8.1). The source code of OralSeg is released under the Apache License 2.0. Users are free to copy, distribute, display, and adapt the model for research, education, and medical training.

## Discussion

This study established a dataset by precisely annotating 35 anatomical structures in dental CBCT and used it to train a deep learning model, OralSeg, which is suited for CBCT tooth instance segmentation. The model integrated the strengths of SwinViT and spatial Mamba and performed multi-scale residual fusion during decoding to extract key anatomical features for multi-class tooth segmentation. The results showed that OralSeg significantly enhanced the performance of deep learning models in recognizing and segmenting complex oral anatomical structures, particularly demonstrating higher accuracy and stability in regions such as apical areas, alveolar bone margins, and mandibular canals. Moreover, the segmentation tool is freely and openly available and is offered in a user-friendly “one-click” format for clinical dentists and researchers without AI development experience.

The advancement of digital healthcare has enabled deep learning-assisted automated medical image segmentation, but instance segmentation of teeth and jawbones remains highly challenging due to their numerous categories, fine anatomical structures, and high spatial complexity [38, 39]. Crowns and restorations, especially fixed bridges, were found to introduce artifacts that complicated tooth-level instance segmentation. These artifacts often blurred boundaries and occasionally caused mis-segmentation. Traditional CNN-based models like 3D U-Net and VNet struggle with densely packed, morphologically similar structures in CBCT images due to limited receptive fields, often causing boundary blurring and segmentation errors [40–42]. While multi-stage methods improve accuracy, they increase complexity and error accumulation [43, 44]. Vision Transformers (ViT) offer global context modeling but require high computational resources and large datasets [45–47]. Mamba (SSM) provides stable long-range modeling via state space mechanisms, but lacks spatial sensitivity and interpretability, limiting its standalone clinical applicability [48, 49].

An ideal model must ensure accurate detection and precise segmentation of each target while minimizing adhesion or overlap between adjacent structures. Therefore, tooth instance segmentation imposes dual requirements on models which are the ability to capture global semantic context and local spatial relationships with structural details. The results of our study indicated that the integration of SwinViT and spatial Mamba modules through sequential modeling can produce a synergistic benefit [50]. On one hand, SwinViT extracts global dependencies from long-range CBCT images, capturing semantic associations between distant structures and enhancing understanding of complex anatomical layouts [51, 52]. On the other hand, the spatial Mamba module uses multi-scale SSM in 3D directions to enhance the perception of local spatial structures, showing higher sensitivity and stability in capturing small targets, boundary continuity, and complex morphological variations [32]. This hybrid architecture not only improves the comprehensiveness of feature representation but also optimizes the balance between model efficiency and segmentation accuracy by enhancing feature reuse. Dice is more sensitive to smaller targets like tooth roots and apices, providing a more balanced assessment when there are fewer target voxels. Xie et al. applied a multi-task CNN to predict tooth foregrounds and center landmarks, followed by marker-controlled watershed to separate overlapping crowns, achieving a DSC of 0.88 [53]. In contrast, IoU has stricter criteria for small target segmentation and is more sensitive to both false negatives and false positives. Lee et al. developed a point-based CBCT tooth instance segmentation framework by regressing heatmaps and boxes to separate adjacent teeth, achieving an IoU of 0.704 [54]. OralSeg exhibits stronger Dice and IoU in complex 3D image segmentation tasks, showing the advantage in segmenting detailed anatomical structures such as teeth and bones. In addition, OralSeg exhibits better generalization, and demonstrates higher stability, making it suitable for deployment in clinical environments.

The reported accuracy values were notably high, which measures the proportion of correctly predicted voxels to total voxels, including both true positives and true negatives, which means it includes the impact of a large amount of background information. In dental CBCT images, voxel distribution is highly imbalanced, with bone-related labels occupying a large proportion of the volume. Such a large amount of background and labels imbalance can inflate the overall accuracy, as correctly predicting the dominant background or large structures substantially increases the metric, even if smaller structures are less accurately segmented. Similar high accuracy values have also been reported in previous studies. Cui et al. achieved a detection accuracy of 0.9955% in the first application of deep learning to dental CBCT segmentation [25], and Gerhardt et al. reported a general accuracy of 0.997% when assessing an AI-driven tool for automated detection of teeth and edentulous regions in CBCT images [40]. For these reasons, and following a medical image segmentation evaluation guideline [55], the Dice was adopted as the primary metric for performance interpretation, with additional metrics and visualization included to provide a more comprehensive and comparable evaluation.

Manual segmentation of dental CBCT images often takes several hours [56]. It is highly valuable to make a “one-click” automatic segmentation tool using deep learning techniques in clinical settings to improve workflow efficiency. Dot et al. [33] developed DentalSegmentator, which achieved automatic segmentation of dentition, jawbones, and mandibular canals. Although it made progress in overall structure recognition, it still could not achieve fine segmentation of individual teeth. In this study, we developed and implemented OralSeg, integrating it into the 3D Slicer platform with a complete graphical user interface, allowing clinical users to load CBCT scans and obtain instance segmentation results without programming experience. To accommodate different computing capabilities, users can flexibly define the ROI, balancing model accuracy with computational resource constraints. Additionally, the system supports CBCT image selection, one-click segmentation execution, intuitive 3D reconstruction visualization, and post-processing or manual refinement, enabling users to quickly adjust results based on practical needs. This study not only addresses a gap in the current research field but also significantly enhances tool usability and practicality through user-friendly design, reducing the manual annotation burden and demonstrating strong potential for clinical translation, medical education, and research applications. We are committed to open and reproducible research, and the related code is publicly available for download. Both training and inference source code, as well as the model weights are publicly available.

There are some limitations to this study. Although the integration of SwinViT and the spatial Mamba module significantly enhanced instance segmentation performance, the hybrid architecture results in increased GPU memory consumption and still requires further optimization [57]. Moreover, due to the current implementation of the Mamba module, deployment is restricted to Linux-based environments. In addition, the CBCT scans used in this study were from a single source. Further validation on diverse datasets is necessary to ensure consistent performance across different equipment, imaging settings, and patient populations. Researchers can locally deploy the model with pretrained weights or retrain it with private datasets to enhance its applicability. Future work will continue to iteratively refine the framework by embedding ethical constraints and risk-averse caution modules to deliver a more efficient, generalizable, and interpretable tooth instance segmentation system that strengthens patient safety and trust in medical AI [58, 59].

## Conclusions

In this study, the deep-learning model, OralSeg demonstrated good performance and robustness in segmenting anatomical regions such as jawbones, teeth, and mandibular canals in CBCT imaging. It was subsequently integrated into 3D Slicer extension with a user-friendly graphical interface, enabling one-click segmentation, allowing dental clinicians and researchers without AI expertise to use it conveniently for diagnosis, teaching, and research.

## Supplementary Information

Below is the link to the electronic supplementary material.

Supplementary Material 1 (DOCX 450 KB)

## Data Availability

Code on Github: [https://github.com/OttoYouZhou/oralseg](https:/github.com/OttoYouZhou/oralseg); 3D Slicer extension code on Github: [https://github.com/mihaitarce/SlicerOralImplantTools](https:/github.com/mihaitarce/SlicerOralImplantTools).

## References

[1] Barriviera M, Duarte WR, Januario AL, Faber J, Bezerra AC (2009) A new method to assess and measure palatal masticatory mucosa by cone-beam computerized tomography. J Clin Periodontol 36:564–568. 10.1111/j.1600-051X.2009.01422.x 19538329 10.1111/j.1600-051X.2009.01422.x

[2] White SC, Yoon DC, Tetradis S (1999) Digital radiography in dentistry: what it should do for you. J Calif Dent Assoc 27:942–952 10726560

[3] Casiraghi M, Scarone P, Bellesi L, Piliero MA, Pupillo F, Gaudino D, Fumagalli G, Del Grande F, Presilla S (2021) Effective dose and image quality for intraoperative imaging with a cone-beam CT and a mobile multi-slice CT in spinal surgery: a phantom study. Phys Med 81:9–19. 10.1016/j.ejmp.2020.11.006 33310424 10.1016/j.ejmp.2020.11.006

[4] Issrani R, Prabhu N, Sghaireen MG, Ganji KK, Alqahtani AMA, Alanazi TSAL, Alanazi AM, Alam SH MK and, Munisekhar MS (2022) Cone-Beam computed tomography: A new tool on the horizon for forensic dentistry. Int J Environ Res Public Health 19. 10.3390/ijerph19095352 10.3390/ijerph19095352 PMC9104190 35564747

[5] Nasseh I, Al-Rawi W (2018) Cone beam computed tomography. Dent Clin North Am 62:361–391. 10.1016/j.cden.2018.03.002 29903556 10.1016/j.cden.2018.03.002

[6] Adibi S, Zhang W, Servos T, O’Neill PN (2012) Cone beam computed tomography in dentistry: what dental educators and learners should know. J Dent Educ 76:1437–1442 23144478

[7] Jacobs R, Salmon B, Codari M, Hassan B, Bornstein MM (2018) Cone beam computed tomography in implant dentistry: recommendations for clinical use. BMC Oral Health 18:88. 10.1186/s12903-018-0523-5 29764458 10.1186/s12903-018-0523-5 PMC5952365

[8] Patel S, Brown J, Pimentel T, Kelly RD, Abella F, Durack C (2019) Cone beam computed tomography in endodontics - a review of the literature. Int Endod J 52:1138–1152. 10.1111/iej.13115 30868610 10.1111/iej.13115

[9] Horner K, Barry S, Dave M, Dixon C, Littlewood A, Pang CL, Sengupta A, Srinivasan V (2020) Diagnostic efficacy of cone beam computed tomography in paediatric dentistry: a systematic review. Eur Arch Paediatr Dent 21:407–426. 10.1007/s40368-019-00504-x 31858481 10.1007/s40368-019-00504-x PMC7415745

[10] Assiri H, Dawasaz AA, Alahmari A, Asiri Z (2020) Cone beam computed tomography (CBCT) in periodontal diseases: a systematic review based on the efficacy model. BMC Oral Health 20:191. 10.1186/s12903-020-01106-6 32641102 10.1186/s12903-020-01106-6 PMC7341656

[11] Li S, Fevens T, Krzyak A, Jin C, Li S (2007) Semi-automatic computer aided lesion detection in dental X-rays using variational level set. Pattern Recognit 40:2861–2873. 10.1016/j.patcog.2007.01.012

[12] Cui Z, Fang Y, Mei L, Zhang B, Yu B, Liu J, Jiang C, Sun Y, Ma L, Huang J, Liu Y, Zhao Y, Lian C, Ding Z, Zhu M, Shen D (2022) A fully automatic AI system for tooth and alveolar bone segmentation from cone-beam CT images. Nat Commun 13:2096. 10.1038/s41467-022-29637-2 35440592 10.1038/s41467-022-29637-2 PMC9018763

[13] Wang S, Lei C, Liang Y, Sun J, Xie X, Wang Y, Zuo F, Bai Y, Li S, Liu YJ (2024) A 3D dental model dataset with pre/post-orthodontic treatment for automatic tooth alignment. Sci Data 11:1277. 10.1038/s41597-024-04138-7 39580508 10.1038/s41597-024-04138-7 PMC11585566

[14] Ryu J, Kim YH, Kim TW, Jung SK (2023) Evaluation of artificial intelligence model for crowding categorization and extraction diagnosis using intraoral photographs. Sci Rep 13:5177. 10.1038/s41598-023-32514-7 36997621 10.1038/s41598-023-32514-7 PMC10063582

[15] Said EH, Nassar DEM, Fahmy G, Ammar HH (2006) Teeth segmentation in digitized dental x-ray films using mathematical morphology. IEEE Trans Inf Forensics Secur 1:178–189. 10.1109/tifs.2006.873606

[16] Nardi C, Molteni R, Lorini C, Taliani GG, Matteuzzi B, Mazzoni E, Colagrande S (2016) Motion artefacts in cone beam CT: an in vitro study about the effects on the images. Br J Radiol 89:20150687. 10.1259/bjr.20150687 26577438 10.1259/bjr.20150687 PMC4985217

[17] Wang H, Minnema J, Batenburg KJ, Forouzanfar T, Hu FJ, Wu G (2021) Multiclass CBCT image segmentation for orthodontics with deep learning. J Dent Res 100:943–949. 10.1177/00220345211005338 33783247 10.1177/00220345211005338 PMC8293763

[18] Barandiaran I, Macía I, Berckmann E, Wald D, Dupillier MP, Paloc C, Graña M (2009) An automatic segmentation and reconstruction of mandibular structures from CT-Data. Book title.

[19] Modi CK, Desai NP (2011) A simple and novel algorithm for automatic selection of ROI for dental radiograph segmentation. Book title.

[20] Jiang B, Zhang S, Shi M, Liu H-L, Shi H (2022) Alternate level set evolutions with controlled switch for tooth segmentation. IEEE Access 10:76563–76572. 10.1109/access.2022.3192411

[21] Friedli L, Kloukos D, Kanavakis G, Halazonetis D, Gkantidis N (2020) The effect of threshold level on bone segmentation of cranial base structures from CT and CBCT images. Sci Rep. 10.1038/s41598-020-64383-9 32355261 10.1038/s41598-020-64383-9 PMC7193643

[22] Gan Y, Xia Z, Xiong J, Zhao Q, Hu Y, Zhang J (2016) Toward accurate tooth segmentation from computed tomography images using a hybrid level set model. Med Phys 42:14–27. 10.1118/1.4901521 10.1118/1.4901521 25563244

[23] Evain T, Ripoche X, Atif J, Bloch I (2017) Semi-automatic teeth segmentation in Cone-Beam computed tomography by graph-cut with statistical shape priors. Book title.

[24] Indraswari R, Kurita T, Arifin AZ, Suciati N, Astuti ER, Navastara DA (2018) 3D region merging for segmentation of teeth on Cone-Beam computed tomography images. Book title.

[25] Cui Z, Li C, Wang W (2019) ToothNet: automatic tooth instance segmentation and identification from cone beam CT images. Book title.

[26] He K, Gkioxari G, Dollar P, Girshick R (2017) Mask R-CNN. Book title 10.1109/TPAMI.2018.2844175 29994331

[27] Ezhov M, Zakirov A, Gusarev M (2019) Coarse-to-fine volumetric segmentation of teeth in cone-beam Ct. Book title.

[28] Milletari F, Navab N, Ahmadi S-A (2016) V-Net. Fully Convolutional Neural Networks for Volumetric Medical Image Segmentation. Book title.

[29] Tarce M, Zhou Y, Antonelli A, Becker K (2024) The application of artificial intelligence for tooth segmentation in CBCT images: a systematic review. Appl Sci. 10.3390/app14146298

[30] Hatamizadeh A, Tang Y, Nath V, Yang D, Myronenko A, Landman B, Roth HR, Xu D (2022) UNETR: Transformers for 3D medical image segmentation. Book title.

[31] Liu Z, Lin Y, Cao Y, Hu H, Wei Y, Zhang Z, Lin S, Guo B (2021) Swin transformer: hierarchical vision transformer using shifted windows. Book title.

[32] Xing Z, Ye T, Yang Y, Liu G, Zhu L (2024) SegMamba: Long-Range sequential modeling Mamba for 3D medical image segmentation. Book title. 10.1109/TMI.2025.3589797 40679879

[33] Dot G, Chaurasia A, Dubois G, Savoldelli C, Haghighat S, Azimian S, Taramsari AR, Sivaramakrishnan G, Issa J, Dubey A, Schouman T, Gajny L (2024) Dentalsegmentator: robust open source deep learning-based CT and CBCT image segmentation. J Dent. 10.1016/j.jdent.2024.105130 38878813 10.1016/j.jdent.2024.105130

[34] Tang Y, Yang D, Li W, Roth HR, Landman B, Xu D, Nath V, Hatamizadeh A (2022) Self-Supervised Pre-Training of Swin Transformers for 3D Medical Image Analysis. Book title

[35] Hatamizadeh A, Nath V, Tang Y, Yang D, Roth HR, Xu D (2022) Swin UNETR: Swin Transformers for semantic segmentation of brain tumors in MRI images. Book title.

[36] Çiçek Ö, Abdulkadir A, Lienkamp SS, Brox T, Ronneberger O (2016) 3D U-Net: learning dense volumetric segmentation from sparse annotation. Book title.

[37] Fedorov A, Beichel R, Kalpathy-Cramer J, Finet J, Fillion-Robin JC, Pujol S, Bauer C, Jennings D, Fennessy F, Sonka M, Buatti J, Aylward S, Miller JV, Pieper S, Kikinis R (2012) 3D slicer as an image computing platform for the quantitative imaging network. Magn Reson Imaging 30:1323–1341. 10.1016/j.mri.2012.05.001 22770690 10.1016/j.mri.2012.05.001 PMC3466397

[38] Esteva A, Chou K, Yeung S, Naik N, Madani A, Mottaghi A, Liu Y, Topol E, Dean J, Socher R (2021) Deep learning-enabled medical computer vision. Npj Digit Med. 10.1038/s41746-020-00376-2 33420381 10.1038/s41746-020-00376-2 PMC7794558

[39] Jader G, Fontineli J, Ruiz M, Abdalla K, Pithon M, Oliveira L (2018) Deep instance segmentation of teeth in panoramic X-Ray images. Book title.

[40] Gerhardt MDN, Fontenele RC, Leite AF, Lahoud P, Van Gerven A, Willems H, Smolders A, Beznik T, Jacobs R (2022) Automated detection and labelling of teeth and small edentulous regions on cone-beam computed tomography using convolutional neural networks. J Dent 122:104139. 10.1016/j.jdent.2022.104139 35461974 10.1016/j.jdent.2022.104139

[41] Dou W, Gao S, Mao D, Dai H, Zhang C, Zhou Y (2022) Tooth instance segmentation based on capturing dependencies and receptive field adjustment in cone beam computed tomography. Comput Anim Virtual Worlds. 10.1002/cav.2100

[42] Hsu K, Yuh D-Y, Lin S-C, Lyu P-S, Pan G-X, Zhuang Y-C, Chang C-C, Peng H-H, Lee T-Y, Juan C-H, Juan C-E, Liu Y-J, Juan C-J (2022) Improving performance of deep learning models using 3.5D U-net via majority voting for tooth segmentation on cone beam computed tomography. Sci Rep. 10.1038/s41598-022-23901-7 36396696 10.1038/s41598-022-23901-7 PMC9672125

[43] Khan S, Mukati A, Rizvi SSH, Yazdanie N (2022) Tooth segmentation in 3d cone-beam CT images using deep convolutional neural network. Neural Netw World 32:301–318. 10.14311/nnw.2022.32.018

[44] Shaheen E, Leite A, Alqahtani KA, Smolders A, Van Gerven A, Willems H, Jacobs R (2021) A novel deep learning system for multi-class tooth segmentation and classification on cone beam computed tomography. A validation study. J Dent 115:103865. 10.1016/j.jdent.2021.103865 34710545 10.1016/j.jdent.2021.103865

[45] Shamshad F, Khan S, Zamir SW, Khan MH, Hayat M, Khan FS, Fu H (2023) Transformers in medical imaging: a survey. Med Image Anal 88:102802. 10.1016/j.media.2023.102802 37315483 10.1016/j.media.2023.102802

[46] Lin X, Wang Z, Yan Z, Yu L (2024) Revisiting Self-attention in medical Transformers via dependency sparsification. Book title.

[47] Zhang YH, Huang SC, Zhou ZP, Lungren MP, Yeung S (2022) Adapting Pre-trained vision Transformers from 2D to 3D through weight inflation improves medical image Segmentation. Book title. Jmlr-Journal Machine Learning Research, New Orleans, LA

[48] Salahuddin Z, Woodruff HC, Chatterjee A, Lambin P (2022) Transparency of deep neural networks for medical image analysis: a review of interpretability methods. Comput Biol Med. 10.1016/j.compbiomed.2021.105111 34891095 10.1016/j.compbiomed.2021.105111

[49] Li G, Huang Q, Wang W, Liu L (2025) Selective and multi-scale fusion Mamba for medical image segmentation. Expert Syst Appl. 10.1016/j.eswa.2024.12551840862084

[50] Hatamizadeh A, Kautz J (2024) Mambavision: A hybrid mamba-transformer vision backbone. arXiv preprint arXiv:240708083

[51] Chen J, Mei J, Li X, Lu Y, Yu Q, Wei Q, Luo X, Xie Y, Adeli E, Wang Y, Lungren MP, Zhang S, Xing L, Lu L, Yuille A, Zhou Y (2024) Transunet: rethinking the U-Net architecture design for medical image segmentation through the lens of transformers. Med Image Anal. 10.1016/j.media.2024.103280 39096845 10.1016/j.media.2024.103280

[52] Chen Z, Chen S, Hu F (2023) CTA-UNet: CNN-transformer architecture UNet for dental CBCT images segmentation. Phys Med Biol. 10.1088/1361-6560/acf026 37579767 10.1088/1361-6560/acf026

[53] Xie L, Liu B, Cao Y, Yang C (2022) Automatic individual tooth segmentation in Cone-Beam computed tomography based on Multi-Task CNN and watershed transform. Book title.

[54] Lee J, Chung M, Lee M, Shin Y-G (2022) Tooth instance segmentation from cone-beam CT images through point-based detection and Gaussian disentanglement. Multimedia Tools Appl 81:18327–18342. 10.1007/s11042-022-12524-9

[55] Müller D, Soto-Rey I, Kramer F (2022) Towards a guideline for evaluation metrics in medical image segmentation. BMC Res Notes. 10.1186/s13104-022-06096-y 35725483 10.1186/s13104-022-06096-y PMC9208116

[56] Tang H, Liu S, Shi Y, Wei J, Peng J, Feng H (2025) Automatic segmentation and landmark detection of 3d CBCT images using semi supervised learning for assisting orthognathic surgery planning. Sci Rep. 10.1038/s41598-025-93317-6 40087502 10.1038/s41598-025-93317-6 PMC11909187

[57] Cipriano M, Allegretti S, Bolelli F, Di Bartolomeo M, Pollastri F, Pellacani A, Minafra P, Anesi A, Grana C (2022) Deep segmentation of the mandibular canal: a new 3D annotated dataset of CBCT volumes. IEEE Access 10:11500–11510. 10.1109/access.2022.3144840

[58] Thurzo A (2025) Provable AI ethics and explainability in medical and educational AI agents: trustworthy ethical firewall. Electronics. 10.3390/electronics14071294

[59] Thurzo A, Thurzo V (2025) Embedding fear in medical AI: a risk-averse framework for safety and ethics. Ai. 10.3390/ai6050101

