# Supplementary material for: An open deep learning-based framework and model for tooth instance segmentation in dental CBCT
Source: Clin Oral Investig. 2025 Sep 25;29(10):473. doi: 10.1007/s00784-025-06578-w (PMC12464119; doi:10.1007/s00784-025-06578-w)
Supplement: Supplementary file 1 — Supplementary Material 1 (DOCX 450 KB) [file 784_2025_6578_MOESM1_ESM.docx]

**Supplementary Materials**

**Figure S1. Flowchart of study.**

**
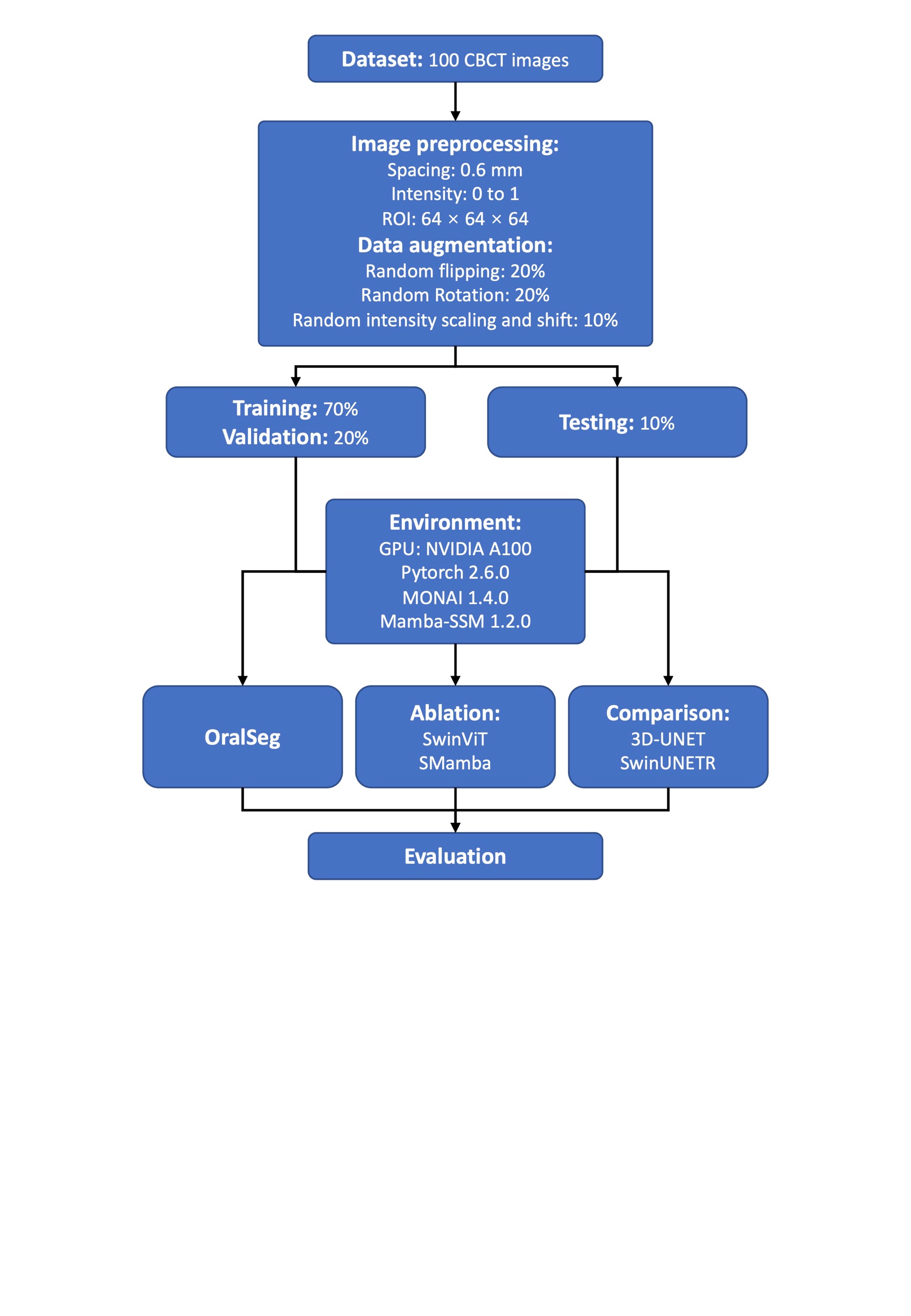
**

**Figure S2. Pairwise ablation model differences in overall Dice (original scale, 95% CI)**

**
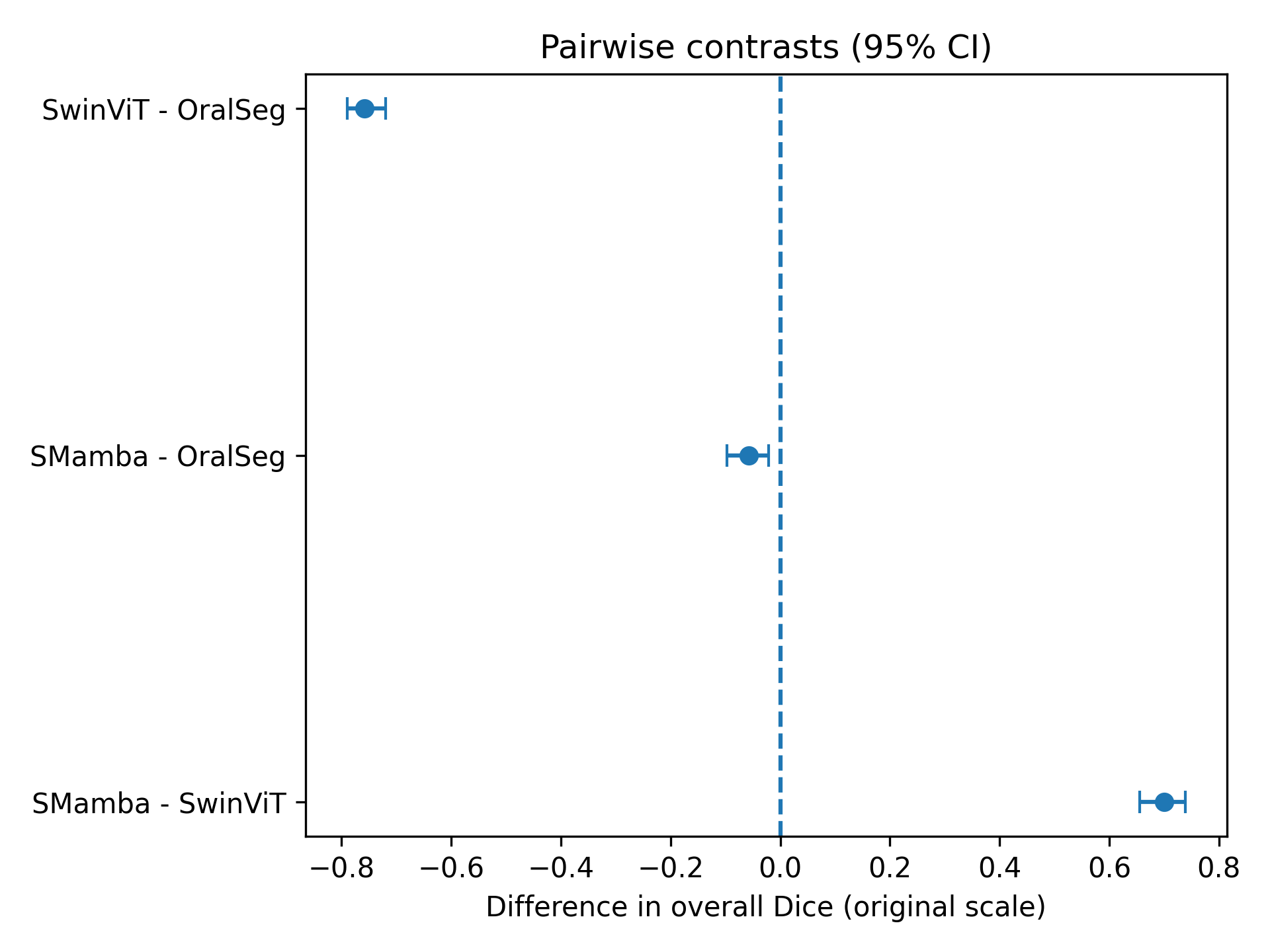
**

**Table S1. Segmentation performance comparison of model and ablation models using different metrics.**

|  | **Accuracy** | **Precision** | **Sensitivity** | **MacroDice** | **IoU** |
| --- | --- | --- | --- | --- | --- |
| **SwinViT** | 0.9994 ± 0.0001 | 0.5440 ± 0.0882 | 0.4956 ± 0.0772 | 0.4831 ± 0.0808 | 0.3854 ± 0.0749 |
| **SMamba** | 0.9996 ± 0.0001 | 0.8174 ± 0.0934 | 0.8080 ± 0.1014 | 0.7983 ± 0.1052 | 0.7111 ± 0.1101 |
| **OralSeg** | 0.9996 ± 0.0001 | 0.8876 ± 0.0265 | 0.8910 ± 0.0338 | 0.8848 ± 0.0322 | 0.8132 ± 0.0363 |

**Table S2. Dataset characteristics: dentition status, third-molar prevalence, and dental restorations.**

| **Variable** | **Value** |
| --- | --- |
| N patients | 100 |
| Fully dentate (28-tooth definition), n (%) | 56 |
| Partially dentate (28-tooth definition), n (%) | 44 |
| Third molars present per patient, n (%) | 1: 14, 2: 15, 3: 14, 4: 37 |
| ≥1 third molar present, n (%) | 80 |
| Dental implants per patient, n (%) | 1: 8, 2: 3 |
| Full-coverage crowns on natural teeth per patient, n (%) | 1: 8, 2: 7, 3: 1, 4: 1, 8: 1 |
| Other restorations on natural teeth per patient, n (%) | 1: 14, 2: 9, 3: 6, 4: 5, 7: 1, 8: 1, 10: 1 |
